# Antiviral activity of animal venom peptides and related compounds

**DOI:** 10.1186/s40409-016-0089-0

**Published:** 2017-01-06

**Authors:** Élida Cleyse Gomes da Mata, Caroline Barbosa Farias Mourão, Marisa Rangel, Elisabeth Ferroni Schwartz

**Affiliations:** 1 Laboratory of Toxinology, Department of Physiological Sciences, University of Brasília, Brasília, DF 70910-900 Brazil; 2 Laboratory of Immunopathology, Butantan Institute, São Paulo, SP 05508-900 Brazil

**Keywords:** Antiretroviral agents, Antiviral agents, HIV, Scorpion venom, Snake venom, Amphibian venom, Insect venom, Marine animal peptides

## Abstract

Viruses exhibit rapid mutational capacity to trick and infect host cells, sometimes assisted through virus-coded peptides that counteract host cellular immune defense. Although a large number of compounds have been identified as inhibiting various viral infections and disease progression, it is urgent to achieve the discovery of more effective agents. Furthermore, proportionally to the great variety of diseases caused by viruses, very few viral vaccines are available, and not all are efficient. Thus, new antiviral substances obtained from natural products have been prospected, including those derived from venomous animals. Venoms are complex mixtures of hundreds of molecules, mostly peptides, that present a large array of biological activities and evolved to putatively target the biochemical machinery of different pathogens or host cellular structures. In addition, non-venomous compounds, such as some body fluids of invertebrate organisms, exhibit antiviral activity. This review provides a panorama of peptides described from animal venoms that present antiviral activity, thereby reinforcing them as important tools for the development of new therapeutic drugs.

## Background

Considering the most common pathologies in humans and other animals, cardiovascular and infectious diseases and cancer are among the leading causes of deaths. The cultural and educational background of affected people largely influences the prevention and treatment of human diseases; nevertheless, the availability of new drugs contributes greatly to mitigating diseases.

More than 200 viruses are known to cause human diseases [1, 2]. Some of them present high public health importance, such as cytomegalovirus (CMV), Epstein-Barr virus (EBV), hepatitis B and C viruses (HBV and HCV, respectively), herpes simplex virus (HSV), human immunodeficiency virus (HIV), rabies virus and Ebola virus. The most recent worldwide estimates presented by the World Health Organization (WHO) reported 1.5 million deaths caused by HIV in 2012, 400 million people living with hepatitis B or C, 80% of liver cancer deaths caused by hepatitis viruses, 500 thousand cases of cervical cancer caused by HPV infection, and over 250 thousand cervical cancer deaths each year [3].

The very few antiviral drugs commercially available can induce severe and considerable adverse effects, especially to those patients receiving lifelong treatment for diseases such as HIV. Furthermore, viruses possess rapid mutational capacity to trick and infect host cells. All these facts together have propelled the prospection for new antiviral drugs, particularly from natural products, as they constitute more than 25% of the new drug prototypes approved in the last decades [4]. Among sources of natural products, animal venoms have revealed a great potential for drug discovery [5–7], and despite the harmful action mechanism of animal venoms, most of them have components holding potential medicinal properties to cure diseases.

It is widely reported in the literature that animal venoms are rich sources of antimicrobial substances, and contain a vast array of active biological compounds with distinct chemical structures [8]. Thus, antimicrobial peptides (AMPs) — a diversified group of peptides that exert essential function in the innate immune host response, when invaded by pathogenic organisms, such as bacteria, fungi and virus — are considered the first line of defense of many organisms, including plants, insects, bacteria and vertebrates [9, 10].

## Possible action mechanism of antiviral compounds

Some peptides exhibit direct virucidal activity; others disturb attachment of virus particles to the cell membrane surface or interfere with the virus replication. Because of the limited efficiency of commonly used drugs and emerging resistance of viruses, antiviral peptides may have the potential for development as putative therapeutic agents [11]. In addition to their reduced market availability, the collateral effects and toxicity of the synthetic antiviral drugs have triggered an expanded search for natural compounds displaying antiviral activities [12, 13]. Any compound to be utilized as an antiviral should comply with the virus pathways during the cellular infectious cycle. Initially, any RNA or DNA virus, enveloped or not, expresses glycoproteins that are responsible for the interaction with surface molecules, receptors, usually glycosylated proteins, integrated in the host cell membrane. At this step, any potential antiviral candidate must compete for the cell receptor by inhibiting the virus attachment to the cell membrane, thereby aborting the viral infection.

**Fig. 1:**
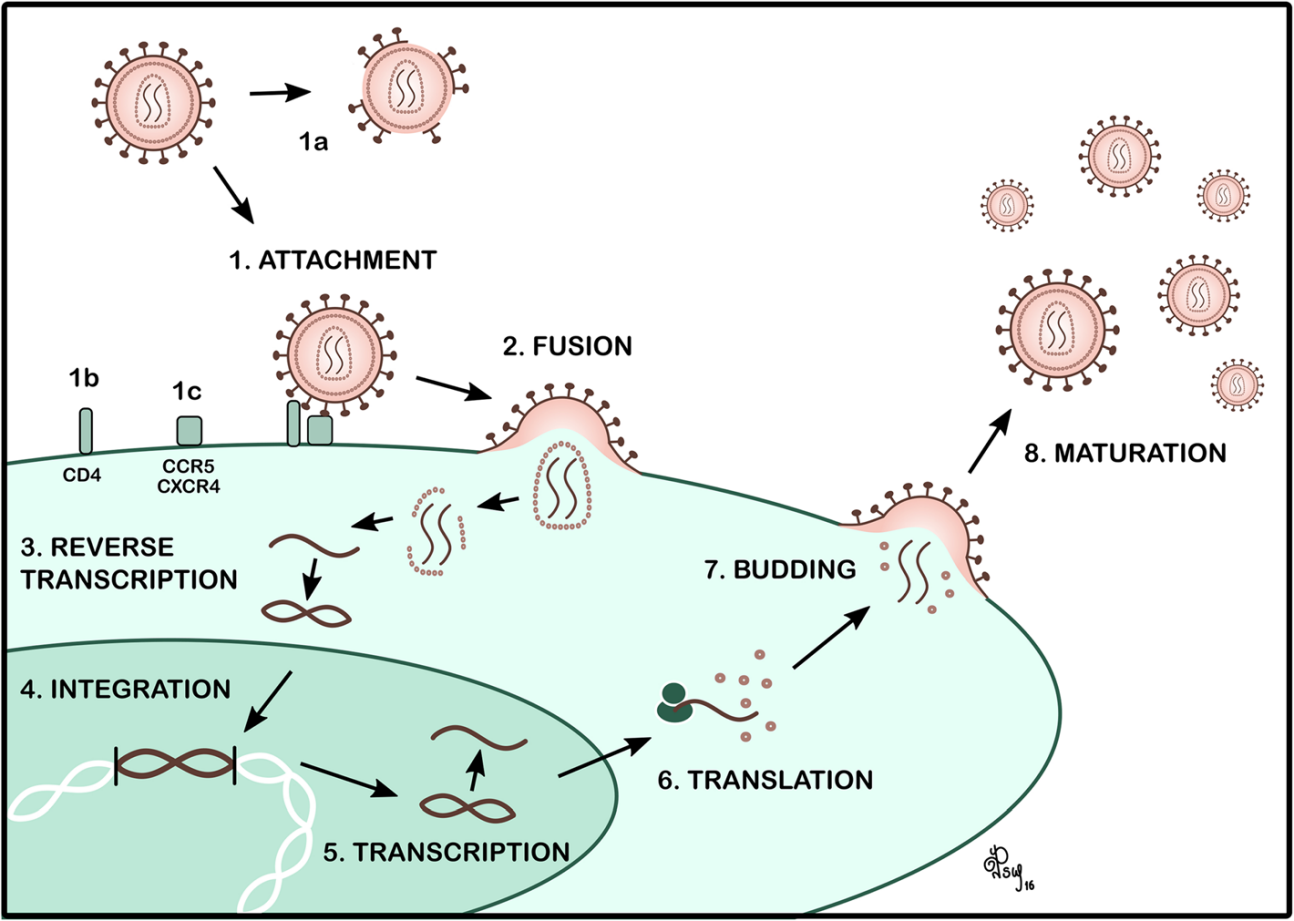
Action mechanism of animal venom peptides or derivatives at different retrovirus replication cycle phases. (**1**) The ChTx and Scyllatoxin-based mimetics, such as CD4M33, inhibit the attachment of the viral glycoprotein (gp120) to the host cell receptor CD4. (**1a**) The peptides cecropin A, magainin 2, papuamide A, dermaseptin DS4, caerins 1.1 and 1.9 and maculation 1.1 disintegrate the viral envelope. (**1b** and **1c**) The peptides CD4M33, BmKn2, Kn2-7, polyphemusin, tachyplesin, immunokine and p3bv obstruct the interaction of the viral gp 120 to the CXCR4 and CCR5 co-receptors. (**2**) The peptides miramides A–H inhibit the fusion of the viral envelope to the host cell membrane. (**3**) The peptides melittin, didemnis A, B and C interfere with the reverse transcription process, aborting the synthesis of double-stranded viral DNA. (**6**) The peptides hecate and TVS-LAO act in the post-translation process, in the cleavage of the GAG/POL protein precursor thus interfering in the assembly of the viral capsid and in the organization of the polymerase complex

Other candidates may act intracellularly by interacting with the virion capsid to prevent its decapsidation; therefore, the viral nucleic acid would not be freed and transcribed. Concerning retroviruses, the antiviral candidates can act by inhibiting (i) the viral reverse transcriptase activity; (ii) the pre-integration complex, thus avoiding the transport of circular viral DNA to the nucleus; (iii) and also by inhibiting the action of the viral integrase, which would not allow the viral DNA to integrate into the cellular chromosome. The proviral DNA, after transcription, is transduced into a polyprotein that requires the viral protease in order to generate small proteins to assemble the viral capsid. In this manner, an antiviral compound could inhibit the viral protease by blocking the retroviral morphogenesis (Fig. 1) [14]. Some retroviral proteins play a major role in the pathogenesis, by down regulation of CD4 and MHC molecules of the host cell, driving them to the proteasome for degradation. If supposed antiviral candidates target these viral proteins, HIV-1 Nef, Tat and Vpr, their actions can be restrained. All the mentioned mechanisms are directly performed by retroviral molecules [15], but other mechanisms could also be triggered, such as those involved in the innate immune system, e.g. (i) the induction of toll-like receptor expression, that interacts with viral nucleic acid, or (ii) production of cytokines that stimulate the action of T cytotoxic cells, and NK cells, and even host cell expression of the major histocompatibility complex molecules, in order to present viral peptides to the other cells of the immune system [16]. Furthermore, antiviral compounds may activate innate restriction factors coded by the host cell [17].

## Mechanisms of viral resistance to drugs

The viral DNA integration in the host cell chromosome represents the major problem to be overcome in a retroviral infection. Until now, there is no available drug capable of completely clearing the virus from the host [18]. Furthermore, silent retroviral infection is hidden at anatomical sites that are difficult to reach by drugs, such as the gut-associated lymphoid tissues, lymph nodes and central nervous system. Infected cells, including macrophages, are quiescent in these tissues and it is not known when they will activate and release new viral progenies. Another challenge for an antiviral candidate is posed by the mutation rate of viral genes, mainly among RNA virus, due to the polymerase synthesis error. This is much more intriguing among retroviruses, as the initial virion genome, maintained in quiescent cells in “sanctuary niche”, are distinct, mutated from each round of cell infection. Thus, in each cycle of viral infection, the hijacked cell produces a growing number of recombinant new virions [19].

## Antiviral peptides obtained from animal venoms

### Scorpion venoms

The arachnid venoms, utilized as a tool for defense and attack, by killing or immobilizing their prey for feeding or their possible competitors and predators, are composed of a rich molecular diversity and complex mixture, with an intricate protein and peptide expression by mechanisms of gene regulation still under investigation [20, 21]. Scorpion venoms have been exhaustively studied, mainly due to the clinical effects after envenomation in humans, which sometimes lead to death [22]. Paradoxically, biotechnological applications are devised by the increased understanding of the action mechanisms of venom components, and therefore, many research works deal with the generation of new drugs based on the structure and function of molecules found in these venoms [23–25].

With the rapid increase in the number of characterized scorpion venom compounds, many new drug candidates have been identified as potential medicines to deal with emerging medical global threats [8, 20]. In scorpions the biologically active peptides are classified as disulfide-bridged peptides (DBPs) and non-disulfide-bridged peptides (NDBPs) [26, 27], with the former being the main components of scorpion venoms, responsible for the neurotoxic symptoms and signs observed during scorpionism. Usually these DBPs target the ion channels of excitable and non-excitable cell membranes. These properties make these molecules interesting prototypes of drugs for the treatment of diverse diseases, particularly those affecting the neural system [8].

In relation to the activity of scorpion venom compounds against retroviruses, such as HIV/SIV, it has been reported that some DBPs can bind to HIV gp120 glycoprotein due to molecular mimicry of lentiviruses host cell CD4^+^ receptor. As a result, they abolish the gp120-CD4 interaction, which is essential to initiate the conformational changes in the viral envelope that trigger viral entry into host cells [28]. These CD4 mimetic scorpion toxins contain about 30 amino acid residues, with three or four disulfide bridges, characterized by the cysteine-stabilized α/β motif (CS-α/β), in which a β-turn between the two β-strands in these peptides resembles the CDR_2_ loop of CD4.

Both charybdotoxin (ChTx) and scyllatoxin, isolated from *Leiurus quinquestriatus hebraeus* venom, present the CS-α/β motif and are capable of blocking K^+^ channels [29–32]. These toxins have been used effectively as molecular scaffolds for gp120-CD4 interaction assays [28, 33, 34]. Since the amino acid residues Phe^43^ and Arg^59^ of CD4 were shown to be critical for CD4 binding to gp120, equivalent amino acid residues were added to the new compounds.

Examples of mimetic peptides using ChTx as a scaffold include CD4M and TXM1, with 33 and 32 amino acid residues, respectively [33, 35]. Among the main modifications, the CD4 CDR_2_ loop sequence ^40^QGSF^43^ was inserted in the equivalent position of the β-turn of ChTx. Thus, Phe^28^ of CD4M, or Phe^27^ of TXM1, would function as Phe^43^ in CD4. The remaining sequence is similar between the two analogs, except in two positions: Arg^20^ in TXM1 (Arg^25^ in ChTx) is replaced by Lys in CD4M, and TXM1 has a Gly^1^ as the N-terminal residue in place of Val^1^-Ser^2^ residues in CD4M. Thus, the charged N-terminus of the Gly^1^ residue in TXM1 is in a position similar to that of the charged side-chain of Arg^59^ in CD4 [33]. CD4M was able to inhibit gp120 binding to CD4 with an IC_50_ value of 20 μM [35]. Likewise, TXM1 also competed with CD4 for gp120 binding, besides causing a CD4-like enhancement in gp120 binding to the antibody 17b [33]. Subsequently, other CD4 mimetics exhibiting gp120 affinity were successfully generated by phage epitope randomization of the β-turn loop in a ChTx-based scaffold [28].

As to scyllatoxin scaffold-based mimetics, a 27-amino acid residue miniprotein named CD4M3 was constructed, which inhibited CD4 binding to gp120 with an IC_50_ value of 40 μM [34]. Structural and functional analysis performed with CD4M3 suggested additional mutations that, once incorporated in the new compound (CD4M9), caused an increased affinity for gp120, with IC_50_ values of 0.1–1.0 μM, depending on the viral strains. Additionally, CD4M9 inhibited infection of CD4^+^ cells by different HIV-1 strains [34]. Its β-turn sequence (^20^AGSF^23^) is similar to that of TXM1. After that, based on CD4M9 structural analysis, a potent mimetic with bona fide CD4-like properties was synthesized [36]. Denominated CD4M33, it inhibited CD4-gp120 binding in different viral strains with 4.0–7.5 nM IC_50_, with these values being comparable to those obtained with CD4. CDM33 also inhibited HIV-1 cell-cell fusion and infection of cells expressing CD4 and either the CCR5 or CXCR4 co-receptors at similar concentrations to CD4 [36]. Its three dimensional structure was further analyzed in complex with gp120 [37]. Then, another analog was designed, denominated F23, which differs from CD4M33 due to the presence of Phe^23^ in replacement by biphenylalanine in position 23 (Bip^23^). The authors showed that F23 had higher mimicry of CD4 than CD4M33. In addition, F23 presented increased neutralization against isolates of phylogenetically related primate lentiviruses [37].

The scorpion venom AMPs belong to NDBPs; many of them and their analogs exert strong antiviral activity, as shown in Table 1. Some of these compounds act by direct rupture of the viral envelope, thereby decreasing viral infectivity [8]. AMPs could also prevent or block the virion from entering into the cell by occupying cell receptors utilized by the viral glycoproteins [38]. Other AMPs do not compete with viral glycoproteins to get attached to cell receptors. Instead, they can cross the cell lipoprotein membrane and internalize themselves in the cytoplasm and organelles, yielding alterations in the profile of host cells that can enhance the defense against the virus or may also block the expression of viral genes in the host cell, halting viral dissemination to other cells [9].

**Table 1:** Scorpion peptides and derivatives with antiviral activity

Scorpion	Peptide name	Virus	EC_50_	Reference
*Lychas mucronatus*	Mucroporin-M1	MeV	7.15 μg/mL (3.52 μM)	[39]
		SARS-CoV	14.46 μg/mL (7.12 μM)	[39]
		H5N1	2.10 μg/mL (1.03 μM)	[39]
		HBV	–	[40]
		HIV-1	–	[38]
*Heterometrus petersii*	Hp1090	HCV	7.62 μg/mL (5.0 μM)	[41]
	Hp1036	HSV-1	0.43 ± 0.09 μM	[42]
	Hp1239	HSV-1	0.41 ± 0.06 μM	[42]
*Mesobuthus martensii*	Kn2-7	HIV-1	2.76 μg/mL (1.65 μM)	[38]
	Bmkn2	HIV-1	–	[38]
*Chaerilus tryznai*	Ctry2459	HCV	1.84 μg/mL	[43]
	Ctry2459-H2	HCV	1.08 μg/mL	[43]
	Ctry2459-H3	HCV	0.85 μg/mL	[43]

*EC*_*50*_ peptide concentration required to reduce virus infection by 50%, *HIV-1* human immunodeficiency virus type 1, *MeV* measles virus, *HBV* hepatitis B virus, *HCV* hepatitis C virus, *SARS-CoV* severe acute respiratory syndrome/coronavirus, *H5N1* influenza virus, *HSV-1* herpes simplex virus type 1. Adapted from Hmed et al. [8]

Mucroporin is a cationic 17-amino-acid residue AMP isolated from *Lychas mucronatus* venom. One of its derivatives, named mucroporin-M1, has an enhanced net positive charge, and besides having antibacterial activity, presented antiviral activity against Measles, SARS-CoV and Influenza H5N1 viruses (Table 1), possibly through a direct interaction with the virus envelope [39]. Additionally, it has been shown to reduce the production of HBV antigens and viral DNA in cell culture microenvironment and also to hinder HBV infection in mouse models [40]. The molecular mechanism implicated reveals the specific activation of mitogen-activated protein kinases (MAPKs) leading to down-regulation of HNF4α expression and consequently less binding to the HBV pre-core/core promoter region [40]. Mucroporin-M1 also presented anti-HIV-1 activity [38].

An amphipathic α-helical peptide, Hp1090, was screened from the cDNA library of *Heterometrus petersii* venomous gland. This 13-amino-acid residue NDBP inhibited the HCV infection (Table 1), acting as a viricide against HCV particles and preventing the initiation of HCV infection by permeabilizing the viral envelope and decreasing virus infectivity [41]. Also from *H. petersii* venom gland cDNA library, other α-helical NDBPs were synthesized. Two of them, Hp1036 and Hp1239, exhibited potent virucidal activity against HSV-1 (Table 1) [42]. They showed inhibitory effects on multiple steps of the virus replication cycle, caused the destruction of the viral morphology and also entered the infected cells where they reduced viral infectivity.

From the cDNA library of *Mesobuthus martensii* venom gland, a compound denominated BmKn2 — with 13 amino acid residues — was cloned and synthesized. Based on its sequence, Kn2-7 was designed by making the substitutions G3K, A4R and S10R, enhancing its net positive charge and α-helix structure [38]. Both compounds exerted anti-HIV-1 activity through inhibition of chemokine receptors CCR5- and CXCR4-mediated activities and replication of the viruses, of which Kn2-7 was the most potent (Table 1) [38].

Another NDBP, screened from *Chaerilus tryznai* scorpion venom gland, Ctry2459, was able to inhibit initial HCV infection in Huh7.5.1 cells by inactivating infectious viral particles (Table 1) [43]. However, due to the low bioavailability of this 13-amino-acid residue peptide, Ctry2459 could not suppress an established infection. Thus, in order to enhance the helicity, amphiphilicity and endosomal escape of peptides, the authors designed histidine-rich peptides based on a Ctry2459 template. Denominated Ctry2459-H2 and Ctry2459-H3, they were more effective against HCV than Ctry2459 (Table 1), significantly reducing intracellular viral production. Unlike Ctry2459, these analogs reduced the viral RNA by 40 and 70%, respectively; however, Ctry2459 diminished viral infectivity in a manner similar to that of wild-type peptide [43].

Recently, the antiviral activities of *Scorpio maurus palmatus* and *Androctonus australis* crude venoms were shown against HCV. They presented IC_50_ values of 6.3 ± 1.6 and 88.3 ± 5.8 μg/mL, respectively. *S. maurus palmatus* venom was considered a good natural source for characterizing new anti-HCV agents targeting the entry step, since it impaired HCV infectivity in cell culture, but not intracellularly, through a virucidal effect. This effect was not inhibited by a metalloprotease inhibitor or heating at 60 °C [44].

### Snake venoms

Snake venoms are composed of a mixture of proteins, peptides (90–95%), free amino acids, nucleotides, lipids, carbohydrates and metallic elements coupled to proteins (5%) [45]. Some studies have reported the antiviral activity of snake venoms and their components against measles virus, Sendai virus, dengue virus (DENV), yellow fever virus (YFV) and HIV [46–50]. Thus, snake venoms are sources of promising candidates for new antiviral drugs (Table 2). In relation to antiretroviral activity, the benefits of treating a patient with multidrug-resistant HIV with a snake venom preparation in addition to the antiretroviral therapy were demonstrated in clinical practice [51]. The response was a decreased viral load and elevated T CD4^+^cell count. The authors suggest that this activity may be related to the presence of some snake venom molecules that are homologous to HIV-1 glycoprotein or proteases [51, 52].

**Table 2:** Examples of animal peptides presenting antiviral activity

Source	Species	Peptide name	Virus	Action mechanism	Reference
Frog	*Xenopus laevis*	Magainin 1 and 2	HSV-1 and HSV-2	Cellular target	[115]
Frog	*Rana brevipodaporsa*	Brevinin-1	HSV	Viral inactivation	[116]
Frog	*Phyllomedusa*	Dermaseptin S_4_	HSV-2	Viral envelope disruption	[69]
Frog	*Phyllomedusa*	Dermaseptin DS_4_	HIV-1	Viral envelope disruption	[117]
Frog	–	Dermaseptin S_4_	HSV-1	Viral membrane disruption	[118]
Frog	*Litoria caerulea*	Caerin 1.1	HIV	Viral envelope disruption	[70]
Frog	*Litoria chloris*	Caerin 1.9	HIV	Viral envelope disruption	[70]
Frog	*Litoria genimaculata*	Maculatin 1.1	HIV	Viral envelope disruption	[70]
Insect	*Vespula lewisii*	MP7-NH_2_	HSV	Viral envelope disruption	[74]
Insect	*Apis mellifera*	Melittin	HIV	CXCR4 and CCR5 tropic inhibition HIV-1 infectivity	[79]
Insect	Synthetic (from melittin)	Hecate	HSV	Cellular target	[83]
Insect	Bee venom	bvPLA_2_	HIV	Virion entry blocking into host cell	[65]
Insect	Synthetic (from bvPLA_2_)	p3bv	HIV	HIV glycoprotein fusion inhibition to CXCR4 cell receptor	[76]
Insect	*Calliphora vicina*	Alloferons 1 and 2	IAV/HSV	Immunomodulatory activity	[88]
Insect	*Hyalophora cecropia*	Cecropin A-magainin 2	HIV	Virion entry blocking into host cell	[85]
Ophidian	*Trimeresurus stejnegeri*	TSV-LAO	HIV-1	Syncytium formation inhibition and HIV-1 p24 antigen reduction	[60]
Ophidian	*Bothrops jararaca*	BjarLAAO-I	DENV-3	Infected cells reduction	[61]
Ophidian	*Crotalus durissus terrificus*	PLA_2_ **-**Cdt	DENV, YFV	Virus envelope cleavage and protein destabilization	[48, 63]
			HIV	Gag p24 processing inhibition	[62, 64]
Ophidian	*Bothrops leucurus*	*Bl*K-PLA_2_; *Bl*D-PLA_2_	DENV	Viral RNA levels reduction	[65]
Ophidian	*Naja kaouthia* (*Naja siamensis*)	Immunokine	HIV	CCR5 and CXCR4 receptors interaction	[7, 66]
Marine sponge	*Sidonops microspinosa*	Microspinosamide	HIV	Cytopathic effect inhibition	[111]
Marine sponge	*Siliquariaspongia mirabilis* and *Stelletta clavosa*	Mirabamides A-H	HIV	Viral glycoprotein fusion neutralization to the cell receptors	[94, 95]
Marine sponge	*Homophymia* sp.	Homophymine A	HIV	Virion entry inhibition	[96]
Marine sponge	*Theonella* sp.	Papuamides A and B	HIV	Virion entry inhibition	[106]
Marine sponge	*Theonella swinhoe*	Theopapuamide A	HIV	Virion entry inhibition	[105, 106]
Marine sponge	*T. swinhoe* and *T. cupola*	Koshikamides F, H	HIV	Virion entry inhibition	[104]
Marine sponge	*Siliquariaspongia mirabilis*	Theopapuamide B	HIV	Viral envelope disruption	[108]
Marine sponge	*Siliquariaspongia mirabilis*	Celebesides A–C	HIV	Virion entry inhibition	[108]
Marine sponge	*Callipelta* sp.	Callipeltin A	HIV	Virion entry inhibition	[109]
Marine sponge	*Neamphius huxleyi*	Neamphamide A	HIV	Virion entry inhibition	[110]
Horseshoe crab	*Tachypleus tridentatus*	Polyphemusin	HIV	Chemokine receptor, CXCR4/viral co-receptor attachment	[112]
Fish	*Pleuronectes americanus*	Pa-MAP	HSV	Viral envelope interaction	[91, 92]
Tunicate	*Trididemnum solidum*	Didemnins A, B and C	HSV-1 and 2; coxsackie virus A-21 and equine rhinovirus	Protein, DNA and RNA synthesis inhibition	[97]

*HIV* human immunodeficiency virus, *HSV* herpes simplex virus, *IAV* influenza virus, *VSV* vesicular stomatitis virus, *DENV* dengue virus. Adapted from Jenssen et al. [9] and Mulder et al. [77]

This homology occurs between the 30–40 highly conserved amino acid residues of snake venom neurotoxins long loop and the sequence 164–174 of short segment HIV-1 gp120. As a result, both may compete for the same receptor or binding site and present anti-HIV activity [50]. The sequence homology between HIV gp120 and snake neurotoxins, such as cobratoxin and bungarotoxin, had generated some antiretroviral patents [53–55]. Linking the gp120 fragment to the HIV peptide fusion inhibitors (fragments of gp41 ectodomains) was shown to improve their anti-HIV efficacy [56]. Besides structural homology, other action mechanisms of snake venoms against HIV are also discussed in the literature, such as catalytic/inhibitory activity through enzymes, binding interference (receptor/enzyme), and induction/interaction at the membrane level [50].

The L-amino acid oxidases (LAAOs or LAOs, EC1.4.3.2), which constitute one of the most studied main components of snake venoms, are oxidoreductase flavoenzymes with molecular masses around 110 to 150 kDa and are usually non-covalently linked homodimeric glycoproteins [57, 58]. These compounds are widely distributed in other organisms and play an important role in biological activities such as apoptosis induction, cytotoxicity, inhibition or induction of platelet aggregation, hemorrhaging, hemolysis and edema, as well as anti-HIV, antimicrobial and antiparasitic activities [59]. TSV-LAO, characterized from *Trimeresurus stejnegeri* snake venom, seems to be the first snake venom LAO reported to present antiviral activity (Table 2) [60].

TSV-LAO is a glycoprotein with a molecular weight of about 58 kDa that also forms homodimers, similarly to LAOs from other snake venoms. Its precursor sequence, obtained by cDNA analysis, codes for a polypeptide of 516 amino acid residues, including an 18-amino-acid potential signal peptide that is identical to those of LAOs from other snake species. TSV-LAO inhibited HIV-1 infection and replication in a dose-dependent manner, and seems to act at nanomolar concentrations by inhibiting syncytium formation (EC_50_ of 1.5 nM) and HIV-1 p24 antigen expression (EC_50_ of 4.1 nM) [60].

Additionally, another LAO, isolated from *Bothrops jararaca* venom and denominated BjarLAAO-I (Table 2), reduced the viral load in cells infected with dengue virus type 3 strain exposed to the toxin in comparison to controls [61]. Its cDNA-deduced sequence has 484 amino acid residues and is similar to other snake venom LAOs. These flavoenzymes also produce hydrogen peroxide (H_2_O_2_) as a free radical, which appears to enhance their antiviral activity [60].

Other compounds found in snake venoms that exhibit antiviral activity are the phospholipases A_2_ (PLA_2_). Among their biological effects, they seem to interact with the host cells and prevent the intracellular release of virus capsid protein, suggesting that they block viral entry into the cells before virion uncoating [7, 49, 62]. The PLA_2_ isolated from *Crotalus durissus terrificus* venom (PLA_2_-Cdt, Table 2) inhibited both DENV and YFV in Vero E6 cells [48]. This PLA_2_ is part of crotoxin, a heterodimeric protein composed of two different subunits non-covalently linked: the basic PLA_2_ (~16.4 kDa) and the acidic protein crotapotin (~9.0 kDa) [48].

The mechanism proposed for PLA_2_-Cdt antiviral activity involves the cleavage of the glycerophospholipid virus envelope and protein destabilization on the virion surface, which partially exposes the genomic RNA and culminates with viral inactivation, making it unable to access the cell receptor [63]. PLA_2_-Cdt also showed in vitro activity against HIV (Table 2) [62, 64], as well as the snake venom PLA_2_s NmmCM_III_ from *Naja mossambica mossambica*, taipoxin from *Oxyuranus scutellatus*, and nigexine from *Naja nigricollis* [49]. Additionally, the PLA_2_ variants, Lys49 and Asp49, denominated *Bl*K-PLA_2_ and *Bl*D-PLA_2_, from *Bothrops leucurus* venom (Table 2), reduced dengue viral RNA in cells treated with these compounds, and presented cytotoxic activity against DENV-infected cells in vitro [65]. *Bl*K-PLA_2_ and *Bl*D-PLA_2_ have 121 and 122 amino acid residues, respectively, including seven disulfide bonds.

Another example of the antiviral effect of biomolecules extracted from snake venoms are the metalloprotease inhibitors, which could prevent the production of new HIV particles by inhibiting the viral proteases [50]. In addition, Immunokine® (OXO Chemie, Thailand), an oxidized derivative of the α-toxin extracted from *Naja siamensis* venom (Table 2), has been shown to inhibit infection of lymphocytes by HIV through the chemokine receptors CCR5 and CXCR4 [7, 66].

### Anuran skin peptides

Many reports detail potent antiviral activity of amphibian skin secretions. Such skin secretions constitute the amphibians’ first line of defense, consisting of their innate immunity. The secretions produced by the anuran skin granular glands have been screened for many biological activities, including antimicrobial, antineoplastic, antiviral, contraceptive and anthelminthic activities [67, 68].

The dermaseptin family of antimicrobial peptides comprise 24–34 amino acids, exhibiting a linear polycationic molecule disposed as an amphiphilic α-helical structure when associated with a lipid cell bilayer. Bergaoui et al. [69] described the dermaseptin S_4_, a chemically synthesized 28-amino-acid drug derived from an amphibian skin antimicrobial peptide, exhibiting anti-herpetic activity (HSV type 2), with reduced cytotoxic effects after biochemical modifications of the original peptide. It also reduced in vitro HIV-1 infection of an established cell line, P4-CCR5, expressing CD4, CCR5, and CXCR4 HIV-1 cell receptors and, primary T lymphocytes, being capable of acting on both R5 and X4 tropic HIV-1 virions. Upon insertion in the viral envelope, the dermaseptin S_4_ disrupts the virion [69].

Caerin 1.1, caerin 1.9 and maculatin 1.1, peptides also derived from the skin secretions of the amphibians *Litoria caerulea*, *Litoria chloris* and *Litoria genimaculata*, respectively, completely abolished HIV infection of T cells, after a few minutes of virion exposure to these modified peptides, which disintegrates the viral envelope, preventing viral fusion to the cell membrane. Furthermore, these molecules obstructed viral transfection from dendritic cells to T cells. Caerin peptides are composed of 25 amino acid residues in their structure, including four central amino acid residues not present in maculatin peptides. In lipid bilayer membranes, these peptides are adjusted to two α-helices, interlinked by a flexible hinge region limited by Pro^15^ and Pro^19^, which determine the disruption of viral envelope and cell membrane [70].

### Insect venoms

Mastoparan is a tetradecapeptide present in wasp (*Vespula lewisii*) venom [71] that forms amphipathic helical structures that insert into lipid bilayers of bacteria, erythrocytes, mast cells and others, forming pores [72, 73]. Mastoparan-7, a mastoparan analogue, displayed a wide spectrum of antiviral activity against enveloped viruses of five different families (Rhabdoviridae, Poxviridae, Flaviridae, Paramyxoviridae and Herpesviridae) in in vitro assays (Table 2). Structural studies have indicated pore formation by the insertion of the mastoporan amphiphilic α helix into the viral lipidic envelope, causing its disruption [74].

HIV virions usually infect the host cells in the genital mucosae, by infecting macrophages, being denominated M-tropic virus; after migrating to the lymph nodes, they infect T lymphocytes, changing into T-tropic virus [75]. Based on the HIV tropism, a phospholipase A_2_ from bee venom, bvPLA_2_, blocked the replication of both M and T-tropic HIV virions [65], while a small peptide derived from bvPLA_2_, the p3bv, exclusively inhibited the replication of T-tropic virus, behaving as a ligand for the HIV-1 co-receptor CXCR4 [49, 76] (Table 2).

AMPs isolated from invertebrate organisms presented augmented antiviral activity in human diseases. Such peptides enclose melittin, cecropin and alloferon molecules [77] (Table 2). Melittin, isolated from honey bee (*Apis mellifera*) venom, is an amphipathic peptide composed of 26 amino acid residues, arranged in two α helical segments. Inserted in nanoparticles, melittin exhibited virucidal activity against HIV-1 in the VK2 cell line, an epithelial vaginal cell line, and also inhibited HIV infection in TZM-bl reporter cells (HeLa cell line expressing HIV receptors) [78–80]. Among other antiretroviral mechanisms, melittin complemented the azidovudin reverse transcription inhibition [81, 82]. Hecate, an analogue of melittin, selectively reduced the protein biosynthesis of virus-specified glycoproteins B, C, D, and H of the HSV type 1 [83]. The mechanism is similar to the one detected among HIV-1 infected lymphoblastic cells, previously treated with melittin, by the intervention in the processing of the gag/pol protein precursor. Therefore, specific intracellular events are targeted by melittin and its derivatives [82, 84].

Cecropins, isolated mostly from the hemolymph of infected pupae of the silk moth *Hyalophora cecropia*, but also from other insects, tunicates and *Ascaris* nematodes, are a family of AMPs, containing 35–37 amino acid residues arranged in two amphiphilic α-helices linked by a Gly-Pro hinge. Synthetic hybrid peptides, namely cecropin A (1–8)-magainin 2 (1–12), exhibited potent antiviral activity by a mechanism mainly based on the compound hydrophobicity and α-helical content, inhibiting the virus-host cell fusion [85] (Table 2).

Alloferon 1 and 2 are peptides constituted of 12–13 amino acid residues, isolated from the hemolymph of the blowfly *Calliphora vicina*. Alloferons exert immunomodulatory activities to control infection by the human influenza virus in mice model of lethal pulmonary infection [75], whereas their derivatives also inhibited in vitro HSV replication in Vero cells [86, 87] (Table 2). These peptides also displayed a relevant role in the innate immunity, being considered prospective peptides for the pharmaceutical industry [88, 89].

### Peptides from marine organisms

Sea organisms are also promising sources of antiviral cationic peptides. They present a broad spectrum of antiviral activity, while one single peptide may present activity against different viruses and other pathogens. The promiscuous antifreeze Pa-MAP peptide, which consists of an α-helix composed of 11 amino acid residues, was isolated from the polar fish *Pleuronectes americanus* (Table 2). The Pa-MAP exerted antimicrobial activity against bacteria, fungi, neoplastic cells, and also interacted with the viral envelope of the HSV types 1 and 2, inhibiting the infection of susceptible cells [77, 90–92].

Some sponge species contain linear or cyclic bioactive peptides composed of atypical amino acid residues, generating unique structures that are rarely found in terrestrial organisms [90, 93]. These compounds, particularly the cyclic depsipeptides mirabamides A-H, isolated from *Siliquaria spongia mirabilis* and *Stelletta clavosa*, obstruct the HIV-1 virion entry into TZM-bl cells, thus neutralizing the viral glycoprotein fusion for expressing CD4 and CCR5 HIV cell receptors [94, 95] (Table 2). Peptide concentrations between 40 and 140 nM were sufficient to inhibit infection by 50% (IC_50_). Another cyclodepsipeptide, homophymine A, obtained from *Homophymia* sp., conferred 50% cell protection at 75 nM concentration against HIV-1 infection in vitro [96] (Table 2).

Discovered in the early 1980s, didemnins A, B and C from the Caribbean tunicate *Trididemnum solidum* were the first antiviral marine depsipeptides described. Didemnins were effective against vaccinia virus, HSV type 1 and 2, coxsackie virus A-21 and equine rhinovirus, presenting strong activity at low doses [97]. Furthermore, these peptides were active in in vivo assays in a rat model infected with herpes simplex virus, reducing the skin lesions after topical administration [98]. Didemnins inhibit protein, DNA and RNA synthesis in cells [99, 100]. The protein synthesis inhibition mechanism may be related to the binding of didemnins to the elongation factor 1 alpha (EF-1 alpha) [101]. Didemnin B underwent phases I and II of clinical trials in the 1980s, but presented low selectivity and therapeutic index, as well as toxic side effects [102]. Dehydrodidemnin B (Aplidin®, Pharma Mar SA, Spain) is currently under phase III of clinical trials as an anticancer drug against multiple myeloma and T-cell lymphoma [103].

Several antiviral peptides and depsipeptides have been described in marine sponges from the genus *Theonella* sp.: koshikamides F and H isolated from *T. swinhoei* and *T. cupola* [104]; papuamides A and B, and theopapuamide A from *Theonella* sp. and *T. swinhoei*, respectively [105–107]. All of them inhibited HIV entry into T cells. Theopapuamide B was isolated from an Indonesian sponge, *Siliquariaspongia mirabilis*, and was also able to inhibit HIV-1 entry into host cells [108]. Papuamide A presented antiviral activity not only against HIV-1, but also against vesicular stomatitis virus and amphotropic murine leukemia virus. Due to its tyrosine residue and the presence of a hydrophobic tail, the peptide may insert into the viral membrane, causing its rupture [105].

Other peptides from marine sponges that inhibit HIV-1 entry into host cells are: callipeltin A, isolated from sponges of the genus *Callipelta*, which displayed antiviral activity with a high selectivity index (29) between the virus and host cells (SI ratio 50% cytotoxic dose [CD_50_]/ED_50_) [109]; celebesides A-C from *Siliquariaspongia mirabilis* [108]; neamphamide A, from *Neamphius huxleyi*, a compound with structural similarities to callipeptins and papuamides that exhibited low toxicity to host cells and a selectivity index above 10 [110]; and microspinosamide, isolated from *Sidonops microspinosa* [111].

Marine arthropod species have also yielded antiviral peptides, tachyplesin and polyphemusin (T140), and shown anti-HIV-1 activity by attachment to the chemokine receptor, CXCR4, which is also the viral T cell co-receptor. Hemocytes of horseshoe crabs (*Tachypleus tridentatus* and *Limulus polyphemus*) are an abundant source of tachyplesin and polyphemusin. The tachyplesin consists of 17–18 amino acid residues, primarily arranged in three tandem repeats of a tetrapeptide, hydrophobic amino acid-Cys-aromatic amino acid-Arg and an amidated C-terminus, while the polyphemusin analog, T140, is composed of 14 amino acid residues, exposing an antiparallel β-sheet conformation stabilized by a disulfide bridge between Cys^4^ and Cys^13^ [112, 113].

## Conclusions

As a consequence of the scarcity of new families of antiviral drugs, pharmaceutical companies have strengthened their efforts to increase developments of known current drugs, resulting in little or even no improvement to the existing therapies. These new patent protections guarantee the rights to the same stakeholders who are charging high consumer prices due to the lack of competition [114]. At the same time, the growing demand for new drugs and natural therapeutic products is a matter of extreme necessity to face the emergency of multiresistant viral pathogens. More than 45 compounds obtained from vertebrate and invertebrate organisms presented in vitro or in vivo antiviral activity. Although none of those has yet been launched on the market as an antiviral drug, they present chemical structures completely different from the current drugs used in therapy, despite acting on similar targets. Those compounds may lead to new classes of therapeutic drugs after additional chemical and pharmacological studies.

Emerging and reemerging viruses of medical relevance challenge health authorities all around the planet. Some viral vaccines have taken too long to be designed and approved for human and animal utilization, and even in some cases could not be developed. Preventive and curative measures should always be in the hands of health authorities to ensure control of epidemics, such as the recent Ebola virus in Africa or arboviruses, particularly in Brazil – represented by the dengue, chikungunya and Zika viruses – or worldwide pandemics, such as influenza and HIV. Therefore, prospection, screening and all other phases of biological activity, validation, clinical development of animal peptides represent an essential scientific investment for protecting and perpetuating humankind.

## Abbreviations

AMP: Antimicrobial peptide
ChTx: Charybdotoxin
CMV: Cytomegalovirus
DBP: Disulfide-bridged peptide
DENV: Dengue virus
EBV: Epstein-Barr virus
H5N1: Influenza virus
HBV: Hepatitis B virus
HCV: Hepatitis C virus
HIV: Human immunodeficiency virus
HSV: Herpes simplex virus
MAPK: Mitogen-activated protein kinase
MeV: Measles virus
NDBP: Non-disulfide-bridged peptide
SARS-CoV: Severe acute respiratory syndrome/coronavirus
VSV: Vesicular stomatitis virus
WHO: World Health Organization
YFV: Yellow fever virus
